# Optineurin Deficiency and Insufficiency Lead to Higher Microglial TDP-43 Protein Levels

**DOI:** 10.3390/ijms23126829

**Published:** 2022-06-19

**Authors:** Nikolina Prtenjaca, Matea Rob, Muhammad S. Alam, Andrea Markovinovic, Cristiana Stuani, Emanuele Buratti, Ivana Munitic

**Affiliations:** 1 Laboratory of Molecular Immunology, Department of Biotechnology, University of Rijeka, R. Matejcic 2, 51000 Rijeka, Croatia; nikolina.prtenjaca@uniri.hr (N.P.); mr852@cam.ac.uk (M.R.); andrea.markovinovic@kcl.ac.uk (A.M.); 2 Department of Medical Genetics, Dementia Research Institute, Cambridge Institute for Medical Research (CIMR), University of Cambridge, Cambridge CB2 0XY, UK; 3 Laboratory of Immune Cell Biology, Center for Cancer Research, National Cancer Institute, NIH, Bethesda, MD 20892, USA; muhammad.alam@nih.gov; 4 Department of Basic and Clinical Neuroscience, Maurice Wohl Clinical Neuroscience Institute, Institute of Psychiatry, Psychology and Neuroscience, King’s College London, London SE5 9RT, UK; 5 International Centre for Genetic Engineering and Biotechnology (ICGEB), Padriciano 99, 34149 Trieste, Italy; cristiana.stuani@icgeb.org (C.S.); emanuele.buratti@icgeb.org (E.B.)

**Keywords:** optineurin, TDP-43, neurodegeneration, amyotrophic lateral sclerosis, neurodegeneration, inflammation, autophagy

## Abstract

Mutations in optineurin, a ubiquitin-binding adaptor protein, cause amyotrophic lateral sclerosis (ALS), a fatal neurodegenerative disease of motor neurons linked to chronic inflammation and protein aggregation. The majority of ALS patients, including those carrying the optineurin mutations, exhibit cytoplasmic mislocalization, ubiquitination, and aggregation of nuclear TAR DNA-binding protein 43 kDa (TDP-43). To address the crosstalk between optineurin and TDP-43, we generated optineurin knockout (KO) neuronal and microglial cell lines using the CRISPR/Cas9 approach. Interestingly, we observed that loss of optineurin resulted in elevated TDP-43 protein expression in microglial BV2 but not neuronal Neuro 2a and NSC-34 cell lines. No changes were observed at the mRNA level, suggesting that this increase was post-translationally regulated. To confirm this observation in primary cells, we then used microglia and macrophages from an optineurin loss-of-function mouse model that lacks the C-terminal ubiquitin-binding region (Optn^470T^), mimicking optineurin truncations in ALS patients. As observed in the BV2 cells, we also found elevated basal levels of TDP-43 protein in Optn^470T^ microglia and bone marrow-derived macrophages. To test if inflammation could further enhance TDP-43 accumulation in cells lacking functional optineurin, we stimulated them with lipopolysaccharide (LPS), and we observed a significant increase in TDP-43 expression following LPS treatment of WT cells. However, this was absent in both BV2 Optn KO and primary Optn^470T^ microglia, which exhibited the same elevated TDP-43 levels as in basal conditions. Furthermore, we did not observe nuclear TDP-43 depletion or cytoplasmic aggregate formation in either Optn^470T^ microglia or LPS-treated WT or Optn^470T^ microglia. Taken together, our results show that optineurin deficiency and insufficiency post-translationally upregulate microglial TDP-43 protein levels and that elevated TDP-43 levels in cells lacking functional optineurin could not be further increased by an inflammatory stimulus, suggesting the presence of a plateau.

## 1. Introduction

Amyotrophic lateral sclerosis (ALS) is a rapidly progressive neurodegenerative disease linked to mutations in >30 different genes or risk factors [1]. Despite wide genetic and functional heterogeneity in ALS patients, chronic neuroinflammation and protein aggregation are two key contributing factors in a rapid loss of motor neurons, paralysis, and consequently death. Although most ALS mutations do not directly target the immune system, microglia, comprising the only resident immune cells of the central nervous system (CNS), become chronically activated and contribute to an excitotoxic environment, neuronal dysfunction, and finally, neuronal death [2,3,4]. In the affected neurons and glia of more than 95% of ALS cases, wild type (WT) TAR DNA-binding protein 43 kDa (TDP-43) is ubiquitinated, hyperphosphorylated, and mislocalized to the cytoplasm [5,6]. TDP-43 is a DNA- and RNA-binding protein involved in multiple steps of RNA metabolism, including mRNA processing, stability, and stress granule formation [7]. It is ubiquitously expressed in a variety of tissues, including the CNS, and is predominantly located in the nucleus. Due to its prion-like C’-terminal domain, TDP-43 is an aggregate-prone protein [8]. Moreover, it is present in motor neurons at a supersaturated state, meaning that it can easily become insoluble [9]. However, the exact reasons for its cytoplasmic mislocalization and aggregation in ALS are still unclear [10,11]. Failure in two main degradation pathways, ubiquitin proteasomal system (UPS) and autophagy, has been investigated as a potential mechanism of mislocalization and aggregation of TDP-43. An analysis of turnover of various TDP-43 variants in cell lines has shown that TDP-43 is primarily degraded by UPS, whereas degradation of aggregated TDP-43 requires autophagy [12,13]. Given that aging is linked to a decline in both UPS and autophagy, this is one of the potential triggers for mislocalization and accumulation of TDP-43 in ALS [13,14]. Another proposed triggering factor for TDP-43 aggregation is inflammation. In line with this, lipopolysaccharide (LPS) treatment induced TDP-43 aggregates in the spinal motor neurons of mice carrying the human TDP-43^A315T^ mutation [15]. There are reports of TDP-43 aggregation in glia as well. Notably, mislocalized and aggregated TDP-43 has been reported in in vitro LPS-treated primary microglia and astrocytes from transgenic TDP-43^A315T^ mice and, to a lesser extent, in WT LPS-treated microglia [16]. However, in comparison to the role of TDP-43 in neurons, its role in glia is still unclear because very few reports have addressed the role of microglial TDP-43 in disease pathogenesis.

Optineurin is a multifunctional adaptor protein whose mutations have been reported in a small percentage of ALS cases [17,18]. Its C’-terminal ubiquitin-binding region is truncated or mutated in the majority of ~40 known ALS-linked mutations, suggesting that ubiquitin binding is necessary for its function. Through its ubiquitin-binding region, optineurin binds to lysine 63 (K63) and methionine 1 (M1) poly-ubiquitin chains, thus facilitating the recruitment of regulatory proteins for several cellular processes, including signal transduction for nuclear factor kappa-light-chain-enhancer of activated B cells (NF-κB) and interferon regulatory factor 3 (IRF3) pathways, vesicle trafficking and/or autophagy [19,20]. Optineurin shares a high level of homology with NF-κB essential modulator (NEMO) in its ubiquitin-binding region; therefore, the initial hypothesis was that it inhibits the NF-κB inflammatory pathway [20]. However, it has been shown in various myeloid cells, including the primary murine microglia, that it is dispensable for NF-κB activation and tumor necrosis factor (TNF) production [21,22,23]. On the contrary, optineurin has been reported as a positive regulator of the IRF3 pathway and interferon-β (IFN-β) production [21,22,23,24,25,26]. Optineurin was also proposed to regulate several membrane-associated trafficking events, including autophagy, which is a key mechanism for lysosome-mediated degradation of ubiquitinated aggregated proteins, damaged organelles, and intracellular pathogens [27,28,29]. Upon binding the ubiquitinated cargo, optineurin bridges it to microtubule-associated protein 1A/1B-light chain-3 (LC3) on autophagosomal membranes, which presents the key event in the autophagy elongation step and formation of autophagosomes. In addition to its role in cargo selection, optineurin has been implicated in two other autophagy steps: the recruitment of autophagy initiation machinery and autophagosome maturation important for lysosome fusion, which is exerted through binding to ubiquitinated myosin VI [28,30]. Therefore, by regulating both inflammation and autophagy, optineurin could participate in the crosstalk between immune signaling and protein clearance.

The autopsies of patients carrying the optineurin mutations have shown TDP-43 aggregation and mislocalization in the spinal and bulbar motor neurons, but the putative direct mechanistic link between these proteins is still elusive [31,32]. It was recently shown that optineurin deficient (Optn^-/-^) mice, as well as ALS patients with homozygous Q398X optineurin truncation, showed TDP-43 cytoplasmic inclusions and multivesicular body protein 2B (CHMP2B) positive vacuoles in the spinal cord motor neurons. The authors proposed that this may be caused by an autophagy block [33]. Similarly, upon overexpression of an aggregate-prone superoxide dismutase 1 (SOD1) G93C ALS patient mutation in HeLa cells, optineurin actively participated in the degradation of SOD1 aggregates [27]. Degradation of TDP-43 aggregates in vitro requires autophagy, and optineurin is recruited to TDP-43 in TDP-43 overexpressing cells [13,34]. Notably, the majority of results on the mechanistic link between optineurin and autophagy were obtained on immortalized cell lines rather than the relevant CNS cells [28,29,35], and few recent studies have been performed in neurons or neuronal cell lines [33,36]. In this study, our main focus was on microglia, in which TDP-43 has been shown to be regulated by inflammation [16]. To this end, we have aimed to analyze how previously characterized optineurin truncation (Optn^470T^) [22], which mimics ALS mutations found in patients, or complete optineurin deficiency generated by the CRISPR/Cas-9 technology affects TDP-43 in primary microglia and related microglial cell line (BV2), respectively.

## 2. Results

### 2.1. Optineurin Deficiency Led to an Increase of TDP-43 in BV2 Microglial Cell Line

**Figure 1:**
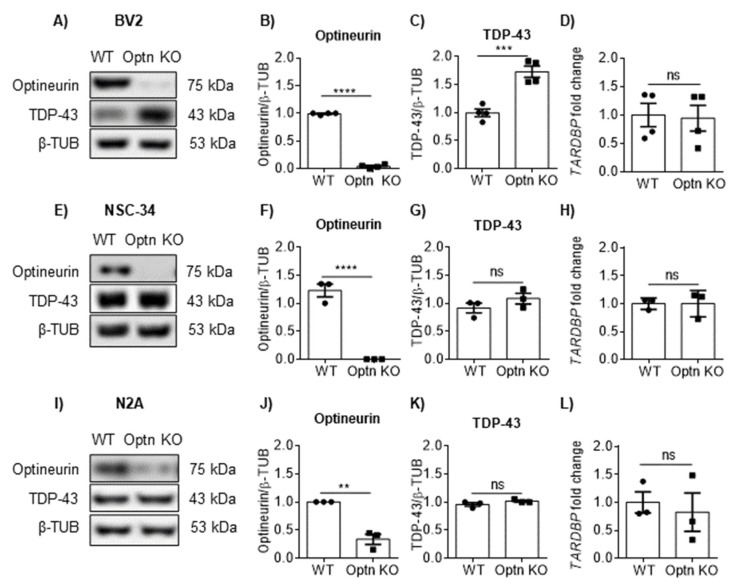
Loss of optineurin leads to an accumulation of TAR DNA-binding protein 43 kDa (TDP-43) protein in microglial but not in neuronal cell lines. (**A**) Western blotting for optineurin and TDP-43 in WT and Optn knockout (KO) microglial cell lines. (**B**,**C**) Bar charts show the densitometric analysis of optineurin and TDP-43 protein levels normalized to β-tubulin in the BV2 cell line. (**D**) mRNA for *TARDBP* in WT and Optn KO BV2 cell lines was assessed by RT-PCR, shown as a difference in fold gene expression (2^−∆∆Ct^) to *GAPDH*. (**E**) Western blotting for optineurin and TDP-43 in WT and Optn KO NSC-34 neuronal cell lines. (**F**,**G**) Bar charts show the densitometric analysis of optineurin and TDP-43 protein levels normalized to β-tubulin in NSC-34. (**H**) mRNA for *TARDBP* in WT and Optn KO NSC-34 cell lines was assessed by RT-PCR. (**I**) Western blotting for optineurin and TDP-43 in WT and Optn KO N2A neuronal cell lines. (**J**,**K**) Bar charts show the densitometric analysis of optineurin and TDP-43 protein levels normalized to β-tubulin in N2A. (**L**) mRNA for *TARDBP* in WT and Optn KO N2A cell lines was assessed by RT-PCR. An average ± SEM from 3 independent experiments is shown. Statistical analysis was performed by Student’s *t*-test: ns, not significant, ** *p* < 0.01, *** *p* < 0.001, **** *p* < 0.0001.

To address the effects of optineurin deficiency on TDP-43 in various brain cell lines, we first established BV2 Optn knockout (KO) microglial, N2A, and NSC-34 neuronal cell lines using CRISPR/Cas9 technology. We confirmed that optineurin was successfully knocked out (with >90% efficiency) in the BV2 microglial cell line (Figure 1A,B). Optineurin was completely deleted in the motor neuron NSC-34 cell line (Figure 1E,F), while in the neuroblastoma N2A cell line, deletion efficiency was around 70% (Figure 1I,J). Optineurin deletion did not affect cell morphology or proliferation of any of these cell lines (data not shown). Upon deletion of optineurin, we compared TDP-43 protein levels and *TARDBP* gene expression between different genotypes in microglial and neuronal cell lines. We observed significantly higher TDP-43 protein levels in BV2 Optn KO cells compared to BV2 WT cells (Figure 1A–C). Notably, the *TARDBP* mRNA levels were not altered in BV2 Optn KO cells (Figure 1D), suggesting that TDP-43 protein levels were post-translationally regulated. In contrast to the microglial BV2 cell line, there was no significant difference in TDP-43 protein or mRNA levels between WT and Optn KO motor neuron NSC-34 cell line (Figure 1E–H). Similarly, we did not observe any difference in TDP-43 protein or mRNA levels in the WT and Optn KO neuroblastoma N2A cell line (Figure 1I–L). Therefore, optineurin deficiency led to the accumulation of TDP-43 protein expression levels in microglial but not neuronal cell lines, and this effect was post-translationally regulated.

### 2.2. Optineurin Insufficiency Led to an Increase of TDP-43 and G3BP1 in Primary Microglia and Macrophages

**Figure 2:**
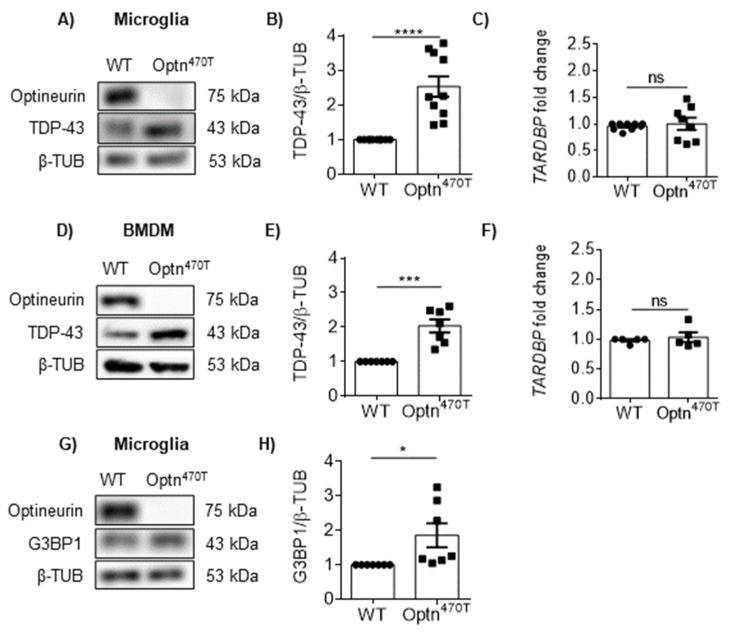
Basal level of TDP-43 is elevated in primary microglia and macrophages carrying the optineurin truncation (Optn^470T^) without changes in mRNA levels. (**A**) Western blotting for TDP-43 and optineurin in WT and Optn^470T^ primary microglia. (**B**) Bar charts show the densitometric analysis of TDP-43 protein levels normalized to β-tubulin in primary microglia. (**C**) mRNA for *TARDBP* in WT and Optn^470T^ microglia was assessed by RT-PCR. (**D**) Western blotting for TDP-43 and optineurin in WT and Optn^470T^ BMDMs. (**E**) Bar charts show the densitometric analysis of TDP-43 protein levels normalized to β-tubulin in BMDMs. (**C**) mRNA for *TARDBP* in WT and Optn^470T^ BMDMs was assessed by RT-PCR. (**G**) Western blotting for G3BP1 in WT and Optn^470T^ primary microglia. (**H**) Bar charts show the densitometric analysis of G3BP1 protein levels normalized to β-tubulin. An average ± SEM from 5 (**F**), 7 (**B**,**H**), 9 (**C**), and 10 (**E**) independent experiments is shown. Statistical analysis was performed by Student’s *t*-test: ns, not significant, * *p* < 0.05, *** *p* < 0.001, **** *p* < 0.0001.

Because our experiments in the BV2 cell line suggested a role of optineurin in the accumulation of TDP-43 protein in microglial cell lines, we next tested TDP-43 levels in primary cells. Most ALS-linked mutations in optineurin are found in the C-terminal ubiquitin-binding region, suggesting that the binding of ubiquitin is crucial for neuroprotection. For this reason, we analyzed the TDP-43 protein levels in the primary neonatal microglia derived from the homozygous Optn^470T^ mice, which lack the C-terminal ubiquitin-binding region. Similar to our observation in BV2 Optn KO microglial cell lines, we found approximately 2-fold higher TDP-43 protein levels in Optn^470T^ microglia compared to WT microglia (Figure 2A,B). In addition, we also did not detect the difference in *TARDBP* mRNA expression (Figure 2C). To see if the increased TDP-43 levels were elevated only in microglia or other myeloid cells as well, we generated the primary macrophages from the bone-marrow precursors from WT and Optn^470T^ mice. We found >2-fold higher TDP-43 protein levels in Optn^470T^ BMDMs, also without a change in *TARDBP* expression (Figure 2D–F), demonstrating that our findings in primary microglia were shared by other myeloid cells. TDP-43 has been shown to positively regulate one of the key proteins in stress granule formation- Ras-GAP SH3-domain-binding protein 1 (G3BP1) [37]. Given that TDP-43 protein levels in Optn^470T^ primary microglia were increased, we next determined whether this impacted the G3BP1 protein levels. We observed more G3BP1 protein levels in Optn^470T^ primary microglia compared to WT cells (Figure 2G,H). Overall, we showed that, similarly to optineurin deficiency in BV2 cells, optineurin insufficiency led to an increased level of TDP-43 and its target G3BP1 in microglia.

### 2.3. Block in Autophagy Is Not the Reason for Increased TDP-43 in BV2 Optn KO and Optn^470T^ Primary Microglia

**Figure 3:**
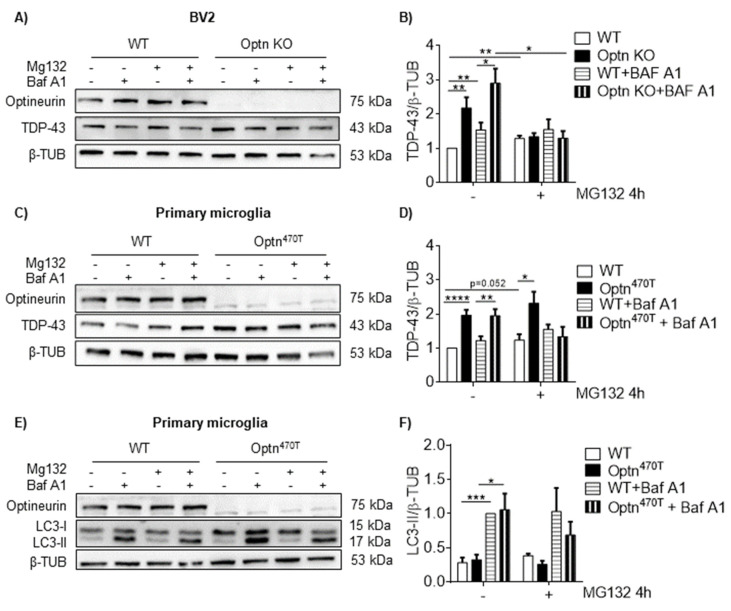
TDP-43 protein accumulation in BV2 Optn KO and Optn^470T^ primary microglia is not caused by a block in autophagy or UPS. (**A**) Western blotting for TDP-43 and optineurin in WT and Optn KO microglial cell lines treated with MG132 and BafA1 for 4 h. (**B**) Bar charts show the densitometric analysis of TDP-43 protein levels normalized to β-tubulin in microglial cells. An average ± SEM from 9 (without MG132) and 4 (with MG132) independent experiments are shown. (**C**) Western blotting for TDP-43 and optineurin in WT and Optn^470T^ primary microglia treated with MG132 and BafA1 for 4 h. (**D**) Bar charts show the densitometric analysis of TDP-43 protein levels normalized to β-tubulin in primary microglia. An average ± SEM from 9 (without MG132) and 4 (with MG132) independent experiments are shown. (**E**) Western blotting for LC3-I, LC3-II, and optineurin in WT and Optn^470T^ primary microglia treated with MG132 and BafA1 for 4 h. (**F**) Bar charts show the densitometric analysis of LC3-II protein levels normalized to β-tubulin in primary microglia. An average ± SEM from 3 independent experiments is shown. Statistical analysis was performed by Student’s *t*-test: * *p* < 0.05, ** *p* < 0.01, *** *p* < 0.001, **** *p* < 0.0001.

To assess the potential mechanism for TDP-43 protein accumulation in the cells lacking functional optineurin, we investigated the two main routes of protein degradation: UPS and autophagy. Autophagy was particularly interesting because of the well-established role of optineurin as an autophagy adaptor [28,29]. In vitro research in the HEK293 cell line has shown that TDP-43 is primarily degraded by UPS, whereas degradation of aggregated (overexpressed) TDP-43 requires autophagy [13]. To evaluate the degradation of TDP-43 in microglia from optineurin deficient and insufficient cells, we blocked UPS by MG132 and lysosomal degradation (the last step in autophagy cascade) by BafA1 and subsequently assessed the TDP-43 protein levels by Western blotting. A short 4 h blockade of either UPS or lysosomal degradation led to a small but significant accumulation of TDP-43 in BV2 WT cells (Figure 3A,B), showing that perhaps 15–25% of TDP-43 was degraded through each of these mechanisms. Simultaneous blockade of both mechanisms in BV2 WT cells did not further increase the TDP-43 levels (Figure 3A,B), suggesting that there was crosstalk between these two disposal systems, as previously observed [13]. No major turnover of optineurin occurred in this time frame by UPS, whereas the turnover by autophagy was small but significant (Supplementary Figure S1A). Of note, MG132 treatments of 8 h or longer led to cell death in both WT and BV2 Optn KO cells. Therefore, we could not analyze the protein turnover at later time points. In BV2 Optn KO cells, which contained a higher level of TDP-43 than WT cells, the blockade of UPS, lysosomes, or both degradation mechanisms together did not increase but rather decreased TDP-43 levels. Although this may have suggested the potential higher toxicity was caused by these blocks in BV2 Optn KO cells, upon evaluating caspase-3 cleavage, we did not see increased apoptosis in Optn KO compared to WT cells (data not shown). Since this peculiarity in degradation could be an artifact of some unknown genetic mutation in BV2 cells, we then tested the same degradation pathways in primary cells. The degradation of TDP-43 by either UPS or lysosomes in a 4 h period was negligible in WT microglia (Figure 3C,D), showing that TDP-43 turnover was slower in these cells than in BV2 cells. The turnover of optineurin in WT cells during the same period was also not substantial, without reaching statistical significance by UPS and lysosomal blockade, and reached significantly higher levels only with combined blockade of both mechanisms (Supplementary Figure S1B). Compared to WT cells, Optn^470T^ microglia showed the same negligible TDP-43 turnover by UPS as observed in WT cells. Notably, the blockade of lysosomal degradation did not change the TDP-43 levels in Optn^470T^ microglia. The blockade of both degradation mechanisms together caused an unexpected drop in TDP-43, similar to the finding in BV2 cells, which we cannot explain at this moment, but as in BV2 cells, we could not detect increased apoptosis in Optn^470T^ cells (data not shown). In parallel, we also analyzed the LC3-I to LC3-II conversion as an autophagosome flux marker. UPS blockade had no effect on LC3-I to LC3-II conversion in any of the genotypes, as expected. In the WT microglia, LC3-II substantially increased upon autophagy blockade (Figure 3E,F), demonstrating active basal autophagy in these cells. Notably, the same increase was observed in Optn^470T^ microglia, demonstrating that optineurin insufficiency did not cause a block in basal autophagy. Given the unexpected lack of autophagy block in optineurin deficiency or insufficiency models of microglia, we also analyzed autophagy flux in primary BMDM. We observed an increase in LC3-II upon blockade of lysosomal degradation in WT BMDM cells (Supplementary Figure S1C,D). The same LC3-II level was observed in Optn^470T^ BMDMs, thus showing that the basal autophagy flux was normal. To conclude, we found that the elevated levels of TDP-43 in primary microglia of Optn^470T^ mice could not be explained by the presence of blockade in either autophagy or UPS. Moreover, by analyzing LC3 conversion, we also did not detect a block in basal autophagy in primary microglia or BMDMs.

### 2.4. LPS Increased TDP-43 in WT Cells, but Failed to Increase the Already Elevated TDP-43 Levels in Optineurin KO and Insufficient Cells

**Figure 4:**
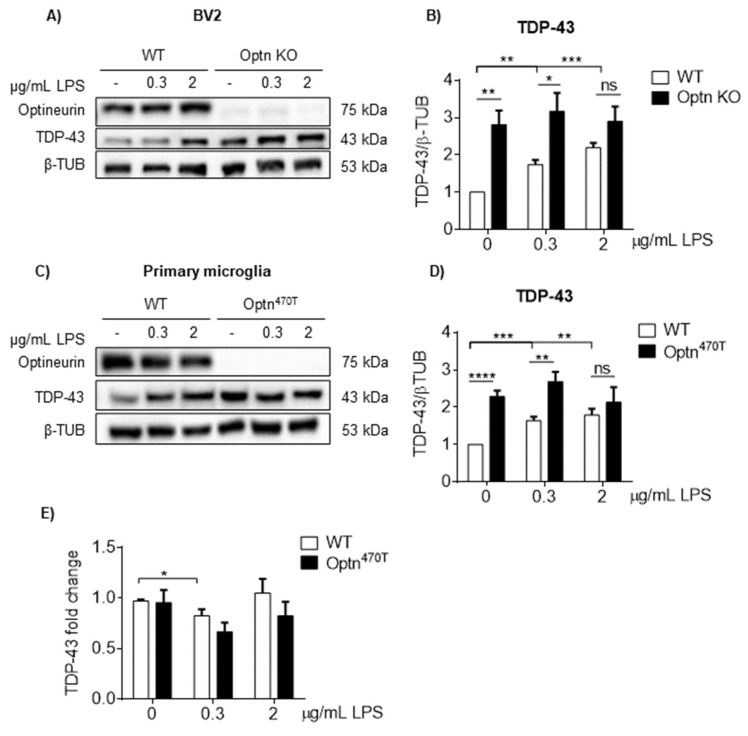
TDP-43 in BV2 Optn KO and Optn^470T^ microglia remains at the same elevated level as in the basal state and does not increase with LPS treatment. (**A**) Western blotting for TDP-43 and optineurin in WT and Optn KO BV2 cell line upon the indicated doses of LPS for 24 h. (**B**) Bar charts show the densitometric analysis of TDP-43 protein levels normalized to β-tubulin in WT and Optn KO BV2 cell lines. (**C**) Western blotting for TDP-43 and optineurin in WT and Optn^470T^ primary microglia upon LPS treatment for 24 h. (**D**) Bar charts show the densitometric analysis of TDP-43 protein levels normalized to β-tubulin in WT and Optn^470T^ primary microglia. (**E**) mRNA for TDP-43 in WT and Optn^470T^ primary microglia was assessed by RT-PCR 24 h upon LPS treatment. An average ± SEM from 5 independent experiments is shown. Statistical analysis was performed by Student’s *t*-test: ns, not significant, * *p* < 0.05, ** *p* < 0.01, *** *p* < 0.001, **** *p* < 0.0001.

Microglia, as the only innate immune cells in the brain parenchyma, are crucial for responding to different pathogen or damage-associated molecular patterns (PAMPs and DAMPs), such as lipopolysaccharide (LPS). Moreover, TDP-43 protein levels are increased in microglia upon LPS treatment [16]. Since we previously showed that optineurin is important in microglial activation and function [21], we wanted to test how the lack of optineurin affects TDP-43 protein levels upon inflammatory stimulation. BV2 WT and Optn KO cell lines, as well as both WT and Optn^470T^ primary microglia, were treated with two different doses of LPS for 24 h, and TDP-43 protein levels were then determined by Western blotting. Similar to the findings of Correia et al., who reported increased TDP-43 protein levels in microglia upon LPS treatment [16], we observed a dose-response increase in TDP-43 levels in the BV2 WT cell line (Figure 4A,B). In contrast, the LPS treatment in BV2 Optn KO cells did not cause any further increase in the already elevated TDP-43 levels found in those cells (Figure 4A,B). The same was true for primary microglia; WT microglia had increased TDP-43 levels in a dose-dependent manner, whereas the TDP-43 levels in Optn^470T^ microglia did not increase from their basal levels (Figure 4C,D). We also analyzed the *TARDBP* mRNA levels and observed no biologically relevant changes in expression between WT and Optn^470T^ primary microglia (Figure 4E), suggesting that the LPS-mediated increase was not transcriptionally regulated. Therefore, the TDP-43 levels reached a plateau in the basal state in BV2 Optn KO and Optn^470T^ microglia, which could not be further exceeded by LPS stimulation.

### 2.5. Increased TDP-43 Does Not Form Aggregates in Microglia

**Figure 5:**
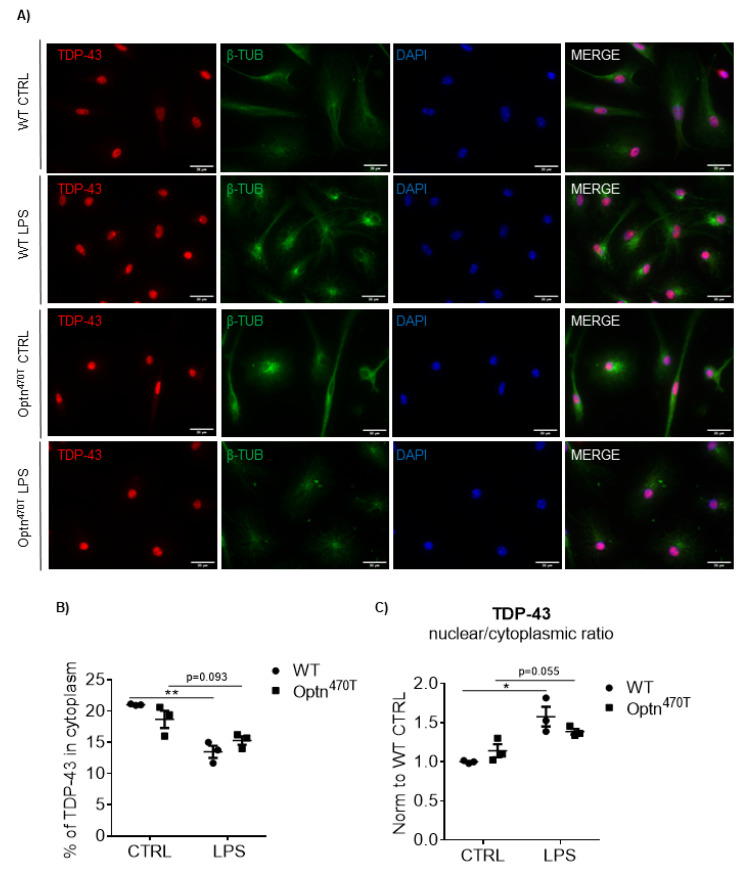
TDP-43 is predominantly nuclear with or without LPS treatment in both WT and Optn^470T^ primary microglia. (**A**) Representative images of immunofluorescence staining for TDP-43 (red), β-tubulin (green), and DAPI (blue) in WT and Optn^470T^ microglia in the basal state and stimulated with 2 µg/mL of LPS for 24 h. (**B**) Graph shows percent of TDP-43 in the cytoplasm in the basal state and upon LPS treatment. (**C**) Nuclear to cytoplasmic ratio of TDP-43 MFI is shown in WT and Optn^470T^ microglia. An average ± SEM from 3 independent experiments is shown. Statistical analysis was performed by Student’s *t*-test: * *p* < 0.05, ** *p* < 0.01. Scale bar = 20 µm.

Since it was reported that upon LPS stimulation, both aggregation-prone mutant and WT TDP-43 are depleted from the nucleus and form cytoplasmic aggregates in microglia [16], we assessed if the elevated levels of TDP-43 found in Optn^470T^ microglia (Figure 2) and LPS-treated WT microglia (Figure 4) caused the redistribution of nuclear TDP-43 to cytoplasm. LPS treatment caused a shape change to ameboid in both WT and Optn^470T^ microglia, demonstrating an efficient activation in both genotypes (Figure 5A). WT microglia in the basal state showed predominantly nuclear TDP-43 staining, with only ~20% of TDP-43 present in the cytoplasm (Figure 5A; quantified in 5B) as previously reported [6]. The elevated TDP-43 levels detected in Optn^470T^ microglia did not result in cytoplasmic TDP-43 accumulation (Figure 5A,B) or its nuclear depletion (Figure 5B,C). In contrast to Correia et al., after 24 h of LPS treatment, we did not observe any cytoplasmic aggregates in WT cells (Figure 5A), but rather found a small drop in the percent of cytoplasmic TDP-43 (Figure 5B), resulting in an increase in the nuclear to cytoplasmic ratio of TDP-43 (Figure 5C). This suggested that the increased TDP-43 found in LPS-treated WT cells (evaluated by immunoblotting in Figure 4) was predominantly nuclear. In comparison to WT cells, LPS-treated Optn^470T^ microglia showed a slightly smaller decrease in the percentage of cytoplasmic TDP-43 and a smaller increase in nuclear to cytoplasmic ratio, which did not reach a statistical significance (*p* = 0.093 and *p* = 0.055, respectively). The latter perhaps suggests that the elevated TDP-43 levels in Optn^470T^ microglia, which did not increase upon LPS treatment (as shown in Figure 4), were even less responsive to cellular redistribution than in WT cells. Importantly, increased TDP-43 observed in Optn^470T^ microglia and LPS-treated WT cells did not cause cytoplasmic TDP-43 accumulation or trigger aggregate formation.

## 3. Discussion

It has been previously shown that autopsy materials of ALS patients carrying optineurin mutations have TDP-43 aggregates and CHMP2B positive autophagic vacuoles in spinal and bulbar motor neurons [31,33]. Although ALS is primarily a disease of motor neurons, it is now well established that microglia are critical triggers of neuropathology in ALS [2,38,39]. To elucidate the potential mechanisms for TDP-43 aggregation and neurodegeneration in ALS patients that carry optineurin mutations, we generated optineurin targeting constructs and silenced this gene using a conventional CRISPR/Cas9 approach. Importantly, we found that following gene expression silencing, the basal levels of TDP-43 protein expression were elevated in the microglia cell lines but, intriguingly, not in neuronal N2A (neural crest-derived neuroblastoma) and NSC-34 (motor neuron-like) cell lines. This was potentially a very interesting observation because cell-specific effects were previously reported in conditional optineurin-deficient mice, whereby the lack of optineurin in oligodendrocytes and microglia, but not neurons and astrocytes, led to a phenotype of axonal dysmyelination [40]. Therefore, we followed up the TDP-43 phenotype of BV2 Optn KO cells in primary microglia from our Optn^470T^ mice model, which lacks the ubiquitin-binding region, akin to the C-terminal truncations found in ALS patients carrying optineurin mutations [22]. Similar to optineurin-deficient BV2 cells, primary Optn^470T^ microglia displayed increased TDP-43 protein levels but not *TARDBP* mRNA. This demonstrated that optineurin deficiency and insufficiency caused by impaired ubiquitin-binding had the same phenotype of TDP-43 increase and that in microglia the expression of TDP-43 is post-translationally affected by the presence of functional optineurin.

Genetic mutations in the components of UPS and autophagy degradation pathways and age-associated decline in these systems have been investigated as potential mechanisms that lead to TDP-43 aggregation [14,41]. Importantly, although all neuronal TDP-43 aggregates are marked by lysine 48 (K48), a substantial proportion of them is simultaneously marked by K63 and linear poly-ubiquitin chains, which can be recognized by optineurin during the process of autophagy [42]. The roles of ALS-linked null optineurin mutations or the mutations directly targeting the ubiquitin-binding region of optineurin, which recognizes K63 and linear poly-ubiquitin chains, have been reported during the autophagy of cytosolic bacteria, protein aggregates, and damaged mitochondria [27,28,29,43]. For this reason, we assessed if the increased TDP-43 levels in BV2 KO cells and Optn^470T^ primary microglia were due to impaired autophagy. However, we found that microglial TDP-43 is not turned over by autophagy, suggesting that it is not present in insoluble aggregates, which we subsequently confirmed by immunofluorescent microscopy. Indeed, despite having increased TDP-43 levels, Optn^470T^ primary microglia did not exhibit aggregates, and TDP-43 was still predominantly nuclear. Moreover, after analyzing basal autophagy by monitoring the LC3-I to LC3-II conversion, we saw active basal autophagy in microglia but found that optineurin or its ubiquitin-binding function was dispensable for this process. Notably, optineurin itself was also not substantially degraded via lysosomes, suggesting that it did not act as an autophagy adaptor for the process of basal autophagy in microglia. Surprised by this finding, we analyzed another myeloid cell type—macrophages—and found that Optn^470T^ BMDM also had increased TDP-43 levels but that their basal autophagy flux was normal. These data are important because the role of optineurin in autophagy has not been uniformly described in the current literature. For example, while ubiquitin-binding optineurin mutations Optn^478G^ and Optn^D474N^ failed to clear the overexpressed mutant TDP-43 in N2A neuronal cell line through autophagy, an ALS patient carrying a heterozygous mutation Optn^478G^ did not test positive for TDP-43-positive autophagic vacuoles, unlike the patient carrying the homozygous Optn^Q398X^ mutation who did [33,43]. Curiously, the most prevalent normal-tension glaucoma-associated optineurin E50K mutation was reported to form insoluble TDP-43 protein aggregates and showed autophagy inhibition [44], although the mechanism of action of glaucoma mutations is thought to be gain-of-function, unlike the loss-of-function ALS mutations. Thus, our results reinforce the conclusion that the role of optineurin in autophagy could differ in various cell types and experimental systems.

Soluble TDP-43 has been shown to be degraded through UPS and has a long half-life [12,13,45]. Curiously, it is still unclear if WT TDP-43 or ALS-linked patient mutations have faster turnover since opposite results were reported in different experimental systems [12,45]. Here, we confirmed that TDP-43 also had a slow turnover in primary microglia, showing that after 4 h of proteasomal blockade, there was <20% increase of TDP-43 in WT cells, and perhaps even less in Optn^470T^, but we could not precisely determine the half-life because higher incubation times with MG132 were toxic. We also did not observe cleavage fragments of TDP-43 (data not shown). Therefore, although we still do not understand the exact mechanism of TDP-43 increase in optineurin-deficient and -insufficient cells, we did not see a significantly diminished degradation by UPS in those cells compared to WT cells.

Since there is evidence for both gain- and loss-of-function of TDP-43 in cellular and animal ALS models, it is evident that TDP-43 optimally functions in a very narrow concentration window [46]. Fittingly, its levels were reported to be tightly autoregulated through a negative feedback mechanism [47,48]. Briefly, TDP-43 binds to 3′UTR of its own mRNA, triggering its alternative splicing, destabilization, and degradation [48]. Because of such autoregulation, heterozygous TDP-43^+ /-^ mice have been reported to have *TARDBP* mRNA and TDP-43 protein levels equal to WT mice [49], and overexpressed TDP-43 in SH-SY5Y cells downregulated endogenous TDP-43 to reach the same total TDP-43 levels [12]. Therefore, it was surprising to find approximately 2-fold elevated levels of TDP-43 in primary microglia and macrophages. Notably, this increase was present in the absence of changes in *TARDBP* mRNA expression levels, meaning that the elevated TDP-43 protein levels in our models were post-translationally regulated. We also report an increase in one of the TDP-43 targets-G3BP1, which is a key component of stress granules [37]. Future studies are necessary to follow up on this finding and see if Optn^470T^ primary microglia alterations occur following stress response. In support of this possibility, it has recently been shown that depletion of optineurin in neurons derived from induced pluripotent stem cells leads to delayed stress granule clearance and an increased level of ubiquitinated TDP-43 in stress granules [50].

Given that the common hallmarks of ALS are proteinopathy and chronic neuroinflammation, several publications have looked for eventual direct links between TDP-43 and inflammation. Cytoplasmic TDP-43 mislocalization has been reported in the circulating monocytes of ALS patients, although in the absence of aggregation or ubiquitination [51]. In contrast, primary mouse microglia exhibited increased TDP-43 levels, its cytoplasmic mislocalization, and aggregate formation upon LPS treatment [16]. Similar to this observation, we found increased levels of TDP-43 upon LPS stimulation in BV2 cell lines and primary microglia. However, in contrast to these findings, we could not trigger cytoplasmic mislocalization and/or aggregate formation. As a limitation of our study, we also considered a potential problem with the detection of aggregates in our experimental system since a recent publication reported that some antibodies do not stain the aggregates in human cells [52]. To address this issue, we tested several antibodies and found that only one worked for immunofluorescence in microglia. Nevertheless, we compared the antibodies by Western blotting and observed the same pattern of TDP-43 expression by both antibodies (data not shown). Thus, we cannot explain why LPS causes aggregation in some cases but not in others. Nevertheless, there are other reports that have also observed that TDP-43 has been difficult to aggregate, even in the presence of patient mutations [12]. This has been ascribed to autoregulation of TDP-43, which is thought to prevent proteotoxic situations. Interestingly, in Optn^470T^ primary microglia, LPS could not further increase TDP-43 levels, and they remained at the same elevated level seen in basal conditions. We hypothesize that this plateau is reached because TDP-43 optimally functions in a very narrow concentration window and even if moderately overexpressed it can be very toxic to cells, especially neurons. Our data suggest that this window may be wider in microglia than in neurons and this is for certain an issue to be explored further.

In conclusion, our data suggest that optineurin deficiency and insufficiency post-translationally upregulate microglial TDP-43 protein levels. We see this as approximately 2-fold elevated TDP-43 protein levels in both primary microglia with loss-of-function optineurin mutation and in the BV2 cell line with optineurin deletion. This elevation does not occur via autophagy blockade. Optineurin has previously been implicated in autophagy of protein aggregates, but here we did not observe TDP-43 aggregation. In optineurin deficient or insufficient cells, TDP-43 levels reach a plateau, which cannot be further increased by inflammation. Further studies in in vivo models will demonstrate the potential effects of elevated levels of TDP-43 in Optn^470T^ microglia and/or macrophages. Of note, we have previously shown that acute intraperitoneal LPS application in young adult mice did not elicit differences in microglial activation between WT and Optn^470T^ microglia [21], but it remains to be seen if other (including chronic) stimuli could trigger neuropathology.

## 4. Materials and Methods

### 4.1. Mice

C57BL/6 mice were obtained from Jackson and expanded in the animal facility at the Medical School of the University of Rijeka. Generation of the optineurin truncated Optn^470T^ mouse model has been previously described [22]. Mice used in this study were backcrossed to C57BL/6 genetic background 11 times. For the generation of primary microglia, timed pregnancies of both C57BL/6 (hereafter referred to as wild type, WT) and homozygous Optn^470T^ mice were set to obtain age-matched neonatal pups. The 3-months-old male mice were used for generating primary bone marrow-derived macrophages (BMDMs). Experimental procedures were performed according to the European Communities Council Directive of 24 November 1986 (86/609/EEC). They were approved by the Ethics Committees of the Department of Biotechnology and Medical School of the University of Rijeka and the Ministry of Agriculture of the Republic of Croatia.

### 4.2. Reagents

Antibody to the C-terminus of optineurin (#100000) was purchased from Cayman Chemical (Michigan, MI, USA). The antibodies against TDP-43 N-terminus (#10782-2-AP), TDP-43 C-terminus (#12892-1-AP), and G3BP1 (#66486-1-IG) were from Proteintech (Manchester, UK); anti-LC3 (#PM036) from MBL (Woburn, MA, USA), and anti-β-tubulin (#T8328) from Sigma-Aldrich (St. Louis, MO, USA). Secondary antibodies labeled with horseradish peroxidase (HRP; anti-rabbit #111-035-144 and anti-mouse #115-035-174) were purchased from Jackson (West Grove, PA, USA). Anti-rabbit Alexa Fluor 555 (#A32732) and anti-mouse Alexa Fluor 488 (#A11029) secondary antibodies were from Invitrogen (Carlsbad, CA, USA). Nitrocellulose membranes (#10600001) were from Cytiva (Marlborough, MA, US), protease and phosphatase inhibitor cocktails, Chemiluminescence Blotting Substrate, and SYBR Green I Master Mix were from Roche (Basel, Switzerland). RNeasy Mini Kit was from Qiagen (Hilden, Germany), and the High Capacity cDNA Reverse Transcription Kit was from Applied Biosystems (Foster City, CA, USA). DNAse (#DN25), poly-L-lysine (#P1274), LPS from *E. coli* O111:B4 (#L4391), Bafilomycin A1 (BafA1, #B1793), and 4′,6-diamidino-2-phenylindole (DAPI) were from Sigma-Aldrich (St. Louis, MO, USA). Puromycin (#0240.2) was purchased from Roth (Oberuzwil, Switzerland) and MG132 (#133407-82-6) from Calbiochem (Darmstadt, Germany). Lipofectamine 3000 was from Invitrogen (Carlsbad, CA, USA), and Aqua-Poly/Mount mounting medium (#18606-20) from Polysciences, Inc (Hirschberg an der Bergstrasse, Germany).

### 4.3. Establishing Optn KO Microglial and Neuronal Cell Lines

Optineurin (Optn) knockout (KO) microglial BV2, spinal motor neuron-like NSC-34, and neuroblastoma Neuro2a (N2A) mouse cell lines (kind gift from Dr. J. Kriz) were generated by the CRISPR/Cas9 technology. To this end, the cell lines were transfected with 2 lentiCRISPR plasmids, encoding for optineurin-directed gRNA-guided Cas9 endonuclease and puromycin N-acetyl-transferase (PAC) with Lipofectamine 3000 according to the manufacturer’s instructions. The gRNAs were targeting the third exon, which contains the translational initiation codon, and the following sequences were used: 1.-*forward,* 5′-CACCGGCTGGGGTGAACCATATTGG-3′ and, 1.-*reverse,* 5′-AAACCCAATATGGTTCACCCCAGCC-3′, 2.-*forward,* 5′-CACCGCTGGGGTGAACCATATTGGA-3′ and 2.-*reverse,* 5′-AAACTCCAATATGGTTCACCCCAGC-3′. After 48 h, the transfection medium was removed, replaced with a complete medium, and the selection based on puromycin resistance was introduced. The concentration of puromycin was tested on non-transfected cells, and the lowest concentration that killed 100% of cells was used (5 μg/mL for N2A and 2 μg/mL for BV2 and NSC-34). The efficiency of optineurin deletion was subsequently checked by Western blotting.

### 4.4. Primary Microglia and Bone Marrow-Derived Macrophages (BMDMs) Isolation and Cultivation

Primary microglia were isolated from the brains of neonatal pups (0–3 days postnatally). To avoid contamination with peripheral macrophages, the meninges were carefully removed from the brains under a dissection microscope. The olfactory bulb and cerebellum were removed, and the remaining brain tissue was chopped into small pieces and incubated with 0.125% trypsin for 15 min at 37 °C with 5% CO_2_. Trypsinization was stopped by adding the Dulbecco’s Modified Eagle Medium (DMEM) supplemented with 10% fetal bovine serum (FBS), 2 mM L-glutamine, and antibiotic/antimycotic solution (10,000 U/mL Penicillin, 10 mg/mL Streptomycin, 25 µg/mL Amphotericin B) referred to as complete DMEM, and tissue was triturated in the presence of 625 μg/mL of DNase I. The cell suspensions were then filtered through the 70 µm cell strainers and centrifuged at 500 rpm (110 g) for 5 min. The cell pellets were resuspended into complete DMEM, plated onto a 0.1 mg/mL poly-L-lysine-coated flask, and cultured for 7–10 days, until complete confluence. The medium was changed the following day and subsequently every 2–3 days. Microglia were detached from the astrocyte layer by shaking for 16 h at 120 rpm and 4 h at 300 rpm and seeded onto poly-L-lysine-coated plates, and used for experiments 48 h after seeding. To generate BMDMs, bone marrow was flushed from the femurs and tibias of 3-month-old WT and Optn^470T^ mice and maintained in RPMI 1640 medium supplemented with 10% FBS, 2 mM L-glutamine, antibiotic/antimycotic solution (10,000 U/mL Penicillin, 10 mg/mL Streptomycin, 25 µg/mL Amphotericin B), and 10 mM HEPES referred to as complete RMPI 1640. To differentiate BMDMs, bone marrow was cultured for 5 days in complete RPMI 1640 medium in the presence of 30% L929 cell line supernatant, which produces macrophage colony-stimulating factor (M-CSF). Of note, the L929 cells were cultured in the complete DMEM.

### 4.5. Cell Culture and Treatment

BV2 cell lines, NSC-34 lines, and primary microglia were maintained in complete DMEM. N2A were cultured in complete Eagle’s minimum essential medium (EMEM) supplemented with 10% FBS, 2 mM L-glutamine, 0.1 mM nonessential amino acids (NEAA), 1 M sodium pyruvate, and antibiotic/antimycotic solution (10,000 U/mL Penicillin, 10 mg/mL Streptomycin, 25 µg/mL Amphotericin B). Cells were left untreated or stimulated with 0.3 µg/mL od 2 µg/mL LPS for 24 h. To determine the mechanism for TDP-43 degradation, BV2 microglial cell line and primary microglia were treated with 1 µM MG132 and 50 nM BafA1 for 4 h or with their combination.

### 4.6. Protein Isolation and Western Blot Analysis

The cells were lysed in radioimmunoprecipitation (RIPA) lysis buffer (50 mM Tris, 150 mM NaCl, 0.5% sodium deoxycholate, and 1% Triton X-100) containing protease and phosphatase inhibitors. After 30 min of incubation on ice, lysates were centrifuged at 14,000 rpm for 10 min. Supernatants were collected and mixed with 4× Laemmli buffer (50 mM Tris pH 6.8, 10% glycerol, 2% SDS, 2% 2-mercaptoethanol, and 0.04% bromophenol blue) and heated for 10 min at 95 °C before separating on 12% or 15% polyacrylamide gels (the latter were used only for LC3). All gels were transferred at 100 V to nitrocellulose membranes, with the exception of those that were later blotted for LC3, which were transferred to polyvinylidene fluoride (PVDF) membranes. The membranes were incubated for 1 h with the blocking buffer consisting of 3% bovine serum albumin (BSA) and 0.1% Tween 20 in Tris-buffered saline and immunoblotted with the indicated primary antibodies overnight at 4 °C. After washing 3 times with 0.1% Tween 20 in Tris-buffered saline, membranes were incubated with the indicated secondary antibodies, washed again (as above), and developed with Chemiluminescence Blotting Substrate using ChemiDoc imaging system (Bio-Rad, Hercules, CA, USA). Densitometric analyses were performed using ImageJ software version 1.53r 21 (National Institutes of Health, Bethesda, MD, USA), and TDP-43, optineurin, LC3-II, and G3BP1 protein levels were normalized to loading control (β-tubulin).

### 4.7. Quantitative Real-Time PCR (RT-PCR)

Total RNA was isolated using RNeasy Mini Kit and then transcribed with the High Capacity cDNA Reverse Transcription Kit following the manufacturer’s instructions. RT-PCR was performed with SYBR Green I Master Mix using LightCycler 480 (Roche). *TARDBP* gene that encodes for TDP-43 protein and *G3BP1* gene expression were normalized to the expression of the housekeeping gene, glyceraldehyde 3-phosphate dehydrogenase (*GAPDH*), and shown as a difference in fold gene expression (2^−∆∆Ct^) compared to WT untreated control. The following primers were used: *Gapdh*-forward, 5′-GGTGCTGAGTATGTCGTGGA-3′; reverse, 5′-GTGGTTCACACCCATCACAA-3′; *TARDBP*-5′-CGAGTCCAGAAAACATCTGACC-3′; reverse, 5′-ACACCSTCGCCCATCTATCAT-3′.

### 4.8. Immunofluorescence Analysis

The cells for immunofluorescence analyses were plated onto chamber slides (#177402; LabTek), fixed in paraformaldehyde for 15 min (4% in phosphate-buffered saline (PBS)), and subsequently permeabilized with 0.1% Triton X-100 in PBS for 15 min. The cells were then incubated in a blocking buffer (0.5% BSA in PBS) for 1 h at room temperature and immunostained with anti-TDP-43 and anti-β-tubulin primary antibodies (1:500) O/N at 4°C. After rinsing three times with PBS, cells were incubated with Alexa Fluor 488- and Alexa Fluor 555-conjugated secondary antibodies (dilution 1:1000) for 1 h at room temperature. The nuclei were stained with 0.5 ng/mL DAPI in PBS. The coverslips with the stained cells were mounted on glass slides with the Aqua-Poly/Mount mounting medium. Cells were imaged using 60X objective on an Olympus IX83 microscope (Tokyo, Japan). The nuclear/cytoplasmic TDP-43 and percent (%) of TDP-43 in the cytoplasm were measured using Fiji software. In brief, TDP-43 integrated density (IntDen), calculated as the product of the area and mean fluorescence intensity (MFI), was measured in the whole cell and then separately only in the nucleus; the nuclear IntDent was then subtracted from the whole cell IntDen to get IntDent from the cytoplasm. MFI from the cytoplasm was measured as IntDent divided by the area of the cytoplasm and put in the ratio with the MFI of the nucleus. The cytoplasmic area was marked based on β-tubulin staining and the nuclear area by DAPI.

### 4.9. Statistical Analysis

Statistical analysis was performed by Student’s *t*-test in GraphPad PrismSoftware 6 (San Diego, CA, USA). The values shown represent the means of at least three independent experiments, and a *p*-Value of <0.05 was considered statistically significant.

## Data Availability

Not applicable.

## Supplementary Materials

The following supporting information can be downloaded at: https://www.mdpi.com/article/10.3390/ijms23126829/s1.

## Abbreviations

ALS, amyotrophic lateral sclerosis; BafA1, Bafilomycin A1; BMDMs, bone marrow-derived macrophages; BSA, bovine serum albumin; CHMP2B, multivesicular body protein 2B; CNS, central nervous system; DAMP, damage associated molecular pattern; DMEM, Dulbecco’s Modified Eagle Medium; EMEM, Eagle’s minimum essential medium; FBS, fetal bovine serum; G3BP1, Ras GTPase-activating protein-binding protein 1; GAPDH, glyceraldehyde 3-phosphate dehydrogenase; HRP, horseradish peroxidase; IFN-β, interferon-β; IntDent, integrated density; IRF3, interferon regulatory factor 3; K48, lysine 48; K63, lysine 63; LC3, microtubule-associated protein 1A/1B-light chain-3; LPS, lipopolysaccharide; M-CSF, macrophage colony-stimulating factor; MFI, mean fluorescence intensity; M1, methionine 1; NEAA, nonessential amino acids; NEMO, NF-κB essential modulator; NF-κB, nuclear factor kappa-light-chain-enhancer of activated B cells; Optn, optineurin; PAC, puromycin N-acetyl-transferase; PAMP, pathogen associated molecular pattern; PBS, phosphate-buffered saline; PVDF, polyvinylidene fluoride; RIPA, radioimmunoprecipitation; SOD1, superoxide dismutase 1; TDP-43, TAR DNA-binding protein 43; TNF, tumor necrosis factor; UPS, ubiquitin proteasomal system; WT, wild type.

## References

[1] Hardiman O., Al-Chalabi A., Chio A., Corr E.M., Logroscino G., Robberecht W., Shaw P.J., Simmons Z., van den Berg L.H. (2017). Amyotrophic Lateral Sclerosis. Nat. Rev. Dis. Primers.

[2] Béland L.-C., Markovinovic A., Jakovac H., De Marchi F., Bilic E., Mazzini L., Kriz J., Munitic I. (2020). Immunity in Amyotrophic Lateral Sclerosis: Blurred Lines between Excessive Inflammation and Inefficient Immune Responses. Brain Commun..

[3] Geloso M.C., Corvino V., Marchese E., Serrano A., Michetti F., D’Ambrosi N. (2017). The Dual Role of Microglia in ALS: Mechanisms and Therapeutic Approaches. Front. Aging Neurosci..

[4] Noristani H.N., Sabourin J.C., Gerber Y.N., Teigell M., Sommacal A., dM Vivanco M., Weber M., Perrin F.E. (2015). Brca1 Is Expressed in Human Microglia and Is Dysregulated in Human and Animal Model of ALS. Mol. Neurodegener..

[5] Arai T., Hasegawa M., Akiyama H., Ikeda K., Nonaka T., Mori H., Mann D., Tsuchiya K., Yoshida M., Hashizume Y. (2006). TDP-43 Is a Component of Ubiquitin-Positive Tau-Negative Inclusions in Frontotemporal Lobar Degeneration and Amyotrophic Lateral Sclerosis. Biochem. Biophys. Res. Commun..

[6] Neumann M., Sampathu D.M., Kwong L.K., Truax A.C., Micsenyi M.C., Chou T.T., Bruce J., Schuck T., Grossman M., Clark C.M. (2006). Ubiquitinated TDP-43 in Frontotemporal Lobar Degeneration and Amyotrophic Lateral Sclerosis. Science.

[7] Mackenzie I.R., Rademakers R. (2008). The Role of Transactive Response DNA-Binding Protein-43 in Amyotrophic Lateral Sclerosis and Frontotemporal Dementia. Curr. Opin. Neurol..

[8] Ratti A., Buratti E. (2016). Physiological Functions and Pathobiology of TDP-43 and FUS/TLS Proteins. J. Neurochem..

[9] Ciryam P., Lambert-Smith I.A., Bean D.M., Freer R., Cid F., Tartaglia G.G., Saunders D.N., Wilson M.R., Oliver S.G., Morimoto R.I. (2017). Spinal Motor Neuron Protein Supersaturation Patterns Are Associated with Inclusion Body Formation in ALS. Proc. Natl. Acad. Sci. USA..

[10] Johnson B.S., Snead D., Lee J.J., McCaffery J.M., Shorter J., Gitler A.D. (2009). TDP-43 Is Intrinsically Aggregation-Prone, and Amyotrophic Lateral Sclerosis-Linked Mutations Accelerate Aggregation and Increase Toxicity. J. Biol. Chem..

[11] Prasad A., Bharathi V., Sivalingam V., Girdhar A., Patel B.K. (2019). Molecular Mechanisms of TDP-43 Misfolding and Pathology in Amyotrophic Lateral Sclerosis. Front. Mol. Neurosci..

[12] Araki W., Minegishi S., Motoki K., Kume H., Hohjoh H., Araki Y.M., Tamaoka A. (2014). Disease-Associated Mutations of TDP-43 Promote Turnover of the Protein Through the Proteasomal Pathway. Mol. Neurobiol..

[13] Scotter E.L., Vance C., Nishimura A.L., Lee Y.-B., Chen H.-J., Urwin H., Sardone V., Mitchell J.C., Rogelj B., Rubinsztein D.C. (2014). Differential Roles of the Ubiquitin Proteasome System and Autophagy in the Clearance of Soluble and Aggregated TDP-43 Species. J. Cell Sci..

[14] Aman Y., Schmauck-Medina T., Hansen M., Morimoto R.I., Simon A.K., Bjedov I., Palikaras K., Simonsen A., Johansen T., Tavernarakis N. (2021). Autophagy in Healthy Aging and Disease. Nat. Aging..

[15] Swarup V., Phaneuf D., Dupré N., Petri S., Strong M., Kriz J., Julien J.-P. (2011). Deregulation of TDP-43 in Amyotrophic Lateral Sclerosis Triggers Nuclear Factor ΚB–Mediated Pathogenic Pathways. J. Exp. Med..

[16] Correia A.S., Patel P., Dutta K., Julien J.-P. (2015). Inflammation Induces TDP-43 Mislocalization and Aggregation. PLoS ONE.

[17] Maruyama H., Morino H., Ito H., Izumi Y., Kato H., Watanabe Y., Kinoshita Y., Kamada M., Nodera H., Suzuki H. (2010). Mutations of Optineurin in Amyotrophic Lateral Sclerosis. Nature.

[18] Markovinovic A., Cimbro R., Ljutic T., Kriz J., Rogelj B., Munitic I. (2017). Optineurin in Amyotrophic Lateral Sclerosis: Multifunctional Adaptor Protein at the Crossroads of Different Neuroprotective Mechanisms. Prog. Neurobiol..

[19] Akutsu M., Dikic I., Bremm A. (2016). Ubiquitin Chain Diversity at a Glance. J. Cell Sci..

[20] Laplantine E., Fontan E., Chiaravalli J., Lopez T., Lakisic G., Véron M., Agou F., Israël A. (2009). NEMO Specifically Recognizes K63-Linked Poly-Ubiquitin Chains through a New Bipartite Ubiquitin-Binding Domain. EMBO J..

[21] Markovinovic A., Ljutic T., Béland L.-C., Munitic I. (2018). Optineurin Insufficiency Disbalances Proinflammatory and Anti-Inflammatory Factors by Reducing Microglial IFN-β Responses. Neuroscience.

[22] Munitic I., Giardino Torchia M.L., Meena N.P., Zhu G., Li C.C., Ashwell J.D. (2013). Optineurin Insufficiency Impairs IRF3 but Not NF-ΚB Activation in Immune Cells. J. Immunol..

[23] Slowicka K., Vereecke L., Mc Guire C., Sze M., Maelfait J., Kolpe A., Saelens X., Beyaert R., van Loo G. (2016). Optineurin Deficiency in Mice Is Associated with Increased Sensitivity to *Salmonella* but Does Not Affect Proinflammatory NF-ΚB Signaling. Eur. J. Immunol..

[24] Gleason C.E., Ordureau A., Gourlay R., Arthur J.S.C., Cohen P. (2011). Polyubiquitin Binding to Optineurin Is Required for Optimal Activation of TANK-Binding Kinase 1 and Production of Interferon β. J. Biol. Chem..

[25] Meena N.P., Zhu G., Mittelstadt P.R., Giardino Torchia M.L., Pourcelot M., Arnoult D., Ashwell J.D., Munitic I. (2016). The TBK1-Binding Domain of Optineurin Promotes Type I Interferon Responses. FEBS Lett..

[26] Pourcelot M., Zemirli N., Silva Da Costa L., Loyant R., Garcin D., Vitour D., Munitic I., Vazquez A., Arnoult D. (2016). The Golgi Apparatus Acts as a Platform for TBK1 Activation after Viral RNA Sensing. BMC Biol..

[27] Korac J., Schaeffer V., Kovacevic I., Clement A.M., Jungblut B., Behl C., Terzic J., Dikic I. (2012). Ubiquitin-Independent Function of Optineurin in Autophagic Clearance of Protein Aggregates. J. Cell Sci..

[28] Lazarou M., Sliter D.A., Kane L.A., Sarraf S.A., Wang C., Burman J.L., Sideris D.P., Fogel A.I., Youle R.J. (2015). The Ubiquitin Kinase PINK1 Recruits Autophagy Receptors to Induce Mitophagy. Nature.

[29] Wild P., Farhan H., McEwan D.G., Wagner S., Rogov V.V., Brady N.R., Richter B., Korac J., Waidmann O., Choudhary C. (2011). Phosphorylation of the Autophagy Receptor Optineurin Restricts Salmonella Growth. Science.

[30] Tumbarello D.A., Waxse B.J., Arden S.D., Bright N.A., Kendrick-Jones J., Buss F. (2012). Autophagy Receptors Link Myosin VI to Autophagosomes to Mediate Tom1-Dependent Autophagosome Maturation and Fusion with the Lysosome. Nat. Cell Biol..

[31] Ito H., Nakamura M., Komure O., Ayaki T., Wate R., Maruyama H., Nakamura Y., Fujita K., Kaneko S., Okamoto Y. (2011). Clinicopathologic Study on an ALS Family with a Heterozygous E478G Optineurin Mutation. Acta Neuropathol..

[32] Kamada M., Izumi Y., Ayaki T., Nakamura M., Kagawa S., Kudo E., Sako W., Maruyama H., Nishida Y., Kawakami H. (2014). Clinicopathologic Features of Autosomal Recessive Amyotrophic Lateral Sclerosis Associated with Optineurin Mutation: Autosomal Recessive OPTN-ALS. Neuropathology.

[33] Kurashige T., Kuramochi M., Ohsawa R., Yamashita Y., Shioi G., Morino H., Kamada M., Ayaki T., Ito H., Sotomaru Y. (2021). Optineurin Defects Cause TDP43-Pathology with Autophagic Vacuolar Formation. Neurobiol. Dis..

[34] Wang P. (2017). Acetylation-Induced TDP-43 Pathology Is Suppressed by an HSF1-Dependent Chaperone Program. Nat. Commun..

[35] Wong Y.C., Holzbaur E.L.F. (2014). Optineurin Is an Autophagy Receptor for Damaged Mitochondria in Parkin-Mediated Mitophagy That Is Disrupted by an ALS-Linked Mutation. Cell Biol..

[36] Evans C.S., Holzbaur E.L. (2020). Degradation of Engulfed Mitochondria Is Rate-Limiting in Optineurin-Mediated Mitophagy in Neurons. eLife.

[37] Sidibé H., Khalfallah Y., Xiao S., Gómez N.B., Fakim H., Tank E.M.H., Di Tomasso G., Bareke E., Aulas A., McKeever P.M. (2021). TDP-43 Stabilizes *G3BP1* MRNA: Relevance to Amyotrophic Lateral Sclerosis/Frontotemporal Dementia. Brain.

[38] Brown R.H., Phil D. (2017). Amyotrophic Lateral Sclerosis. N. Engl. J. Med..

[39] De Marchi F., Munitic I., Amedei A., Berry J.D., Feldman E.L., Aronica E., Nardo G., Van Weehaeghe D., Niccolai E., Prtenjaca N. (2021). Interplay between Immunity and Amyotrophic Lateral Sclerosis: Clinical Impact. Neurosci. Biobehav. Rev..

[40] Ito Y., Ofengeim D., Najafov A., Das S., Saberi S., Li Y., Hitomi J., Zhu H., Chen H., Mayo L. (2016). RIPK1 Mediates Axonal Degeneration by Promoting Inflammation and Necroptosis in ALS. Science.

[41] Root J., Merino P., Nuckols A., Johnson M., Kukar T. (2021). Lysosome Dysfunction as a Cause of Neurodegenerative Diseases: Lessons from Frontotemporal Dementia and Amyotrophic Lateral Sclerosis. Neurobiol. Dis..

[42] Nakayama Y., Tsuji K., Ayaki T., Mori M., Tokunaga F., Ito H. (2020). Linear Polyubiquitin Chain Modification of TDP-43-Positive Neuronal Cytoplasmic Inclusions in Amyotrophic Lateral Sclerosis. J. Neuropathol. Exp. Neurol..

[43] Shen W.-C., Li H.-Y., Chen G.-C., Chern Y., Tu P. (2015). Mutations in the Ubiquitin-Binding Domain of OPTN/Optineurin Interfere with Autophagy-Mediated Degradation of Misfolded Proteins by a Dominant-Negative Mechanism. Autophagy.

[44] Zhang S., Shao Z., Liu X., Hou M., Cheng F., Lei D., Yuan H. (2021). The E50K Optineurin Mutation Impacts Autophagy-Mediated Degradation of TDP-43 and Leads to RGC Apoptosis in Vivo and in Vitro. Cell Death Discov..

[45] Ling S.-C., Albuquerque C.P., Han J.S., Lagier-Tourenne C., Tokunaga S., Zhou H., Cleveland D.W. (2010). ALS-Associated Mutations in TDP-43 Increase Its Stability and Promote TDP-43 Complexes with FUS/TLS. Proc. Natl. Acad. Sci. USA..

[46] Buratti E. (2015). Functional Significance of TDP-43 Mutations in Disease. Advances in Genetics.

[47] Ayala Y.M., Zago P., D’Ambrogio A., Xu Y.-F., Petrucelli L., Buratti E., Baralle F.E. (2008). Structural Determinants of the Cellular Localization and Shuttling of TDP-43. J. Cell Sci..

[48] Polymenidou M., Lagier-Tourenne C., Hutt K.R., Huelga S.C., Moran J., Liang T.Y., Ling S.-C., Sun E., Wancewicz E., Mazur C. (2011). Long Pre-MRNA Depletion and RNA Missplicing Contribute to Neuronal Vulnerability from Loss of TDP-43. Nat. Neurosci..

[49] Sephton C.F., Good S.K., Atkin S., Dewey C.M., Mayer P., Herz J., Yu G. (2010). TDP-43 Is a Developmentally Regulated Protein Essential for Early Embryonic Development. J. Biol. Chem..

[50] Kakihana T., Takahashi M., Katsuragi Y., Yamashita S.-I., Sango J., Kanki T., Onodera O., Fujii M. (2021). The Optineurin/TIA1 Pathway Inhibits Aberrant Stress Granule Formation and Reduces Ubiquitinated TDP-43. iScience.

[51] De Marco G., Lomartire A., Calvo A., Risso A., De Luca E., Mostert M., Mandrioli J., Caponnetto C., Borghero G., Manera U. (2017). Monocytes of Patients with Amyotrophic Lateral Sclerosis Linked to Gene Mutations Display Altered TDP-43 Subcellular Distribution. Neuropathol. Appl. Neurobiol..

[52] Fernandes N., Nero L., Lyons S., Ivanov P., Mittelmeier T., Bolger T., Buchan J. (2020). Stress Granule Assembly Can Facilitate but Is Not Required for TDP-43 Cytoplasmic Aggregation. Biomolecules.

