# Supplementary material for: Optineurin Deficiency and Insufficiency Lead to Higher Microglial TDP-43 Protein Levels
Source: Int J Mol Sci. 2022 Jun 19;23(12):6829. doi: 10.3390/ijms23126829 (PMC9224222; doi:10.3390/ijms23126829)
Supplement: Supplementary file 1 [file ijms-23-06829-s001.zip › ijms-1741274-supplementary.pdf]

Supplementary Figure S1.

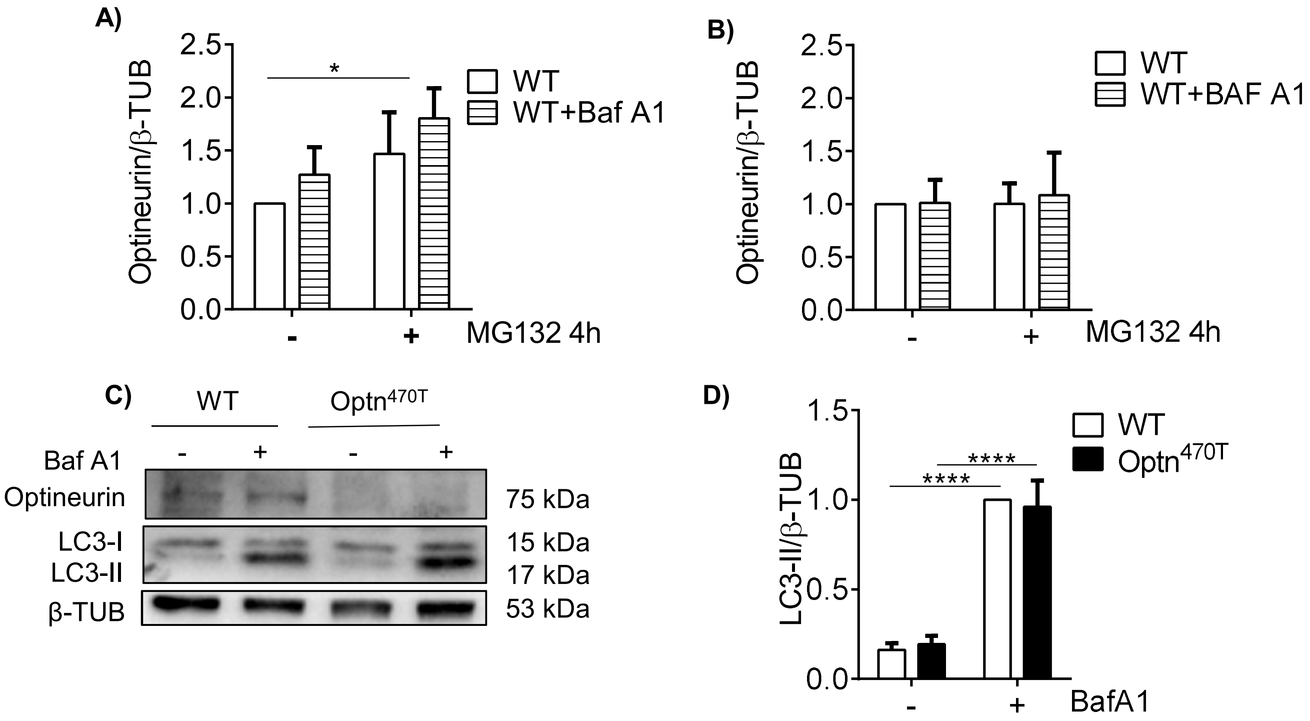

Supplementary Figure S1. UPS and lysosomal degradation blockade in Optn^{470T} primary microglia and macrophages. Bar charts show the densitometric analysis of optineurin protein levels normalized to β-tubulin in (A) WT BV2 cell lines and (B) WT primary microglia treated with MG132 and BafA1 for 4 h. (C) Western blotting for LC3-I and LC3-II in WT and Optn^{470T} BMDMs upon treatment with Baf A1 (200 nM) for 4 h. (D) Bar charts show the densitometric analysis of LC3-II protein levels normalized to β-tubulin in WT and Optn^{470T} BMDMs. An average ± SEM from 4 (A), 5 (B), 7 (C and D) independent experiments is shown. Statistical analysis was performed by unpaired Student's t-test: * $p<0.05$ ** $p<0.01$, **** $p<0.0001$
